# A Sextuple Knockout Cell Line System to Study the Differential Roles of CRY, PER, and NR1D in the Transcription-Translation Feedback Loop of the Circadian Clock

**DOI:** 10.3389/fnins.2020.616802

**Published:** 2020-12-14

**Authors:** Yi-Ying Chiou, Tzu-Ying Li, Yanyan Yang, Aziz Sancar

**Affiliations:** ^1^Graduate Institute of Biochemistry, National Chung Hsing University, Taichung City, Taiwan; ^2^Department of Biochemistry and Biophysics, University of North Carolina School of Medicine, Chapel Hill, NC, United States

**Keywords:** circadian clock, TTFL, Cryptochrome, knockout, serum shock

## Abstract

The transcription-translation feedback loop (TTFL) is the core mechanism of the circadian rhythm. In mammalian cells, CLOCK-BMAL1 proteins activate the downstream genes by binding on the E-box sequence of the clock-controlled genes. Among these gene products, CRY1, CRY2, PER1, PER2, NR1D1, and NR1D2 can regulate the CLOCK-BMAL1-mediated transcription to form the feedback loop. However, the detailed mechanism of the TTFL is unclear because of the complicated inter-regulation of these proteins. Here, we generated a cell line lacking CRY1, CRY2, PER1, PER2, NR1D1, and NR1D2 (Cry/Per/Nr1d_KO) to study TTFL. We compared the Dbp transcription after serum-shock and dexamethasone-shock between Cry/Per/Nr1d_KO cells and cells expressing endogenous CRY (Per/Nr1d_KO) or NR1D (Cry/Per_KO). Furthermore, we found that CRY1-mediated repression of Dbp could persist more than 24 h in the absence of other proteins in the negative limb of the TTFL. Our Cry/Per/Nr1d_KO cells is a suitable system for the studying of differential roles of CRY, PER, and NR1D in the TTFL.

## Introduction

Circadian rhythm is a crucial mechanism that provides a way to adapt to daily environmental changes. Many physiological functions show rhythmic oscillation with the period around 24 h (Bass, 2012; Gumz, 2016). Transcription-translation feedback loop (TTFL) is the core clock required to maintain the circadian rhythm (Reppert and Weaver, 2002; Levi and Schibler, 2007; Hardin and Panda, 2013; Partch et al., 2014). In the mammalian model of TTFL, Circadian Locomotor Output Cycles Kaput protein (CLOCK) and Brain and Muscle ARNT-Like 1 protein (BMAL1) form a heterodimer (CLOCK-BMAL1). CLOCK-BMAL1 binds to the E-box sequence in the promoters of clock-controlled genes and activates their transcription. Among these gene products, Cryptochromes (CRY1/CRY2), Periods (PER1, PER2), and Nuclear Receptor Superfamily 1 Group D (NR1D1/NR1D2) could negatively regulate the activity of CLOCK-BMAL1. CRY binds to CLOCK-BMAL1 on DNA to induce CLOCK-BMAL1-mediated transcriptional repression (Ye et al., 2011, 2014). PER removes CLOCK-BMAL1 from DNA in a CRY-dependent manner to erase the effect of CLOCK-BMAL1 (Ye et al., 2014; Chiou et al., 2016). NR1D binds to the retinoic acid response element (RRE) of the Bmal1 and Cry1 gene to repress their transcription and thus to decrease the CLOCK-BMAL1 and CRY1 levels (Preitner et al., 2002; Ueda et al., 2005).

Studying the biochemical mechanisms of TTFL at the organismal level is challenging. Genetic disruption of these core clock genes may have other developmental problems other than the circadian-related phenotypes. For example, Bmal1 knockout mice show growth retardation, aging, and infertility phenotype (Kondratov et al., 2006). Knockout Nr1d1 also causes postnatal lethality or infertility in C57BL/6 mice (Cho et al., 2012). In another way, too many factors from physiological communications between organs contribute to the rhythmic expression of behavior in mice complicating the data interpretations for mechanism studies. For example, the circadian clock of peripheral tissue could be affected by the neural system and the hormone, which are dynamic and highly regulated (Mohawk et al., 2012). *In vitro* experiments using purified proteins have provided information about the protein-protein interaction (Ye et al., 2011; Xu et al., 2015), DNA binding (Ye et al., 2011), and structures (Huang et al., 2012; Czarna et al., 2013) of the proteins involved in TTFL. However, observations from *in vitro* experiments could not directly link to the transcriptional readouts. Cell lines have been used for the study TTFL because the rhythmic expression of circadian genes could be detected after synchronization (Balsalobre et al., 1998, 2000a). Mouse embryonic fibroblast (MEF) cells from the mice lacking specific TTFL components are suitable tools to study TTFL of the circadian clock. For example, the difference between CRY1 and CRY2 in the maintenance of TTFL has been elucidated using cells from *cry1^–/–^ cry2^–/–^* double knockout mice (Khan et al., 2012). However, the puzzle from the crosstalk between these core clock proteins still could not be excluded. In the case of CRY, changed expression of CLOCK-BMAL1-regulated genes could be interpreted as the change of CRY activity, PER activity, or CLOCK-BMAL1 quantity (through NR1D).

Genome editing approaches provide new strategies to study TTFL of the circadian clock. Cellular studies of TTFL could be performed in the cell lines lacking multiple core clock proteins. Using the MEF cell line lacking CRY and PER (Cry/Per_KO), distinct mechanisms between CRY-mediated CLOCK-BMAL1 inhibition and PER-mediated CLOCK-BMAL1 regulation have been demonstrated (Ye et al., 2014). In another research, the MEF cell line lacking PER and NR1D (Per/Nr1d_KO) was established to study PER-mediated transcriptional activation of CLOCK-BMAL1 regulated genes without the change of CLOCK-BMAL1 level due to the inhibition of Nr1d1 and Nr1d2 by PER (Chiou et al., 2016). These successful cases prompt us to make a cell line model lacking CRY, PER, and NR1D to study TTFL. This cell line would be useful in the study of CLOCK-BMAL1 activity and the study of individual clock proteins in the negative limb of TTFL in a simplified system.

Here, we established a MEF cell line lacking endogenous CRY1, CRY2, NR1D1, NR1D2, PER1, and PER2 proteins (Cry/Per/Nr1d_KO). We performed the RNA-sequencing of Cry/Per/Nr1d_KO cells with Cry/Per_KO and Per/Nr1d_KO cells to compare the effects of endogenous NR1D and CRY on the transcriptome. We also analyzed the transcription of two representative CLOCK-BMAL1-regulated genes, Dbp, and Ciart, after serum or dexamethasone treatment of Cry/Per/Nr1d_KO, Cry/Per_KO, and Per/Nr1d_KO. The data suggested different mechanisms of serum and dexamethasone on the regulation of TTFL. Furthermore, using tamoxifen-controlled CRY1 nuclear localization in Cry/Per/Nr1d_KO cells, we found that CRY-mediated transcriptional repression of Dbp is PER-independent. However, PER is required for the reactivation of Dbp transcription.

## Materials and Methods

### Generation of Mouse Embryonic Fibroblast Cell Line Lacking CRY1, CRY2, PER1, PER2, NR1D1, NR1D2 (Cry/Per/Nr1d_KO)

The Cry1/2^–/–^; Per1/2^–/–^; Nr1d1/2^–/–^ (Cry/Per/Nr1d_KO) MEF was made by CRISPR technology using LentiCRISPRv2 (Sanjana et al., 2014) obtained from Addgene (#52961) to mutate the Nr1d1/2 alleles in Cry1/2^–/–^; Per1/2^–/–^ (Cry/Per_KO) MEF (Ye et al., 2014). The guide RNA sequences for Nr1d1 and Nr1d2 were identical to the sequence making Per1/2^–/–^; Nr1d1/2^–/–^ (Per/Nr1d_KO) MEF (Chiou et al., 2016). Lentivirus for targeting Nr1d1 and Nr1d2 were packaged in HEK293T cells separately and then were mixed for the infection of Cry/Per_KO cells. After puromycin selection, individual colonies were isolated, and the expression of NR1D1 and NR1D2 proteins were analyzed by Western blot. The genomic DNA around the targeting sites were PCR-amplified (primer information was provided in the Supplementary Table) and cloned into a plasmid for sequencing. After successfully isolating the Cry/Per/Nr1d_KO cells, cells were maintained in the DMEM medium supplement with 10% fetal bovine serum (FBS) without puromycin.

### RNA-Sequencing Analysis of Cry/Per/Nr1d_KO, Per/Nr1d_KO, and Cry/Per_KO Cells

Cells were grown in the DMEM medium supplemented with 10% fetal bovine serum at 37°C and 5% CO_2_. Total RNA was prepared when the cells were around 80% confluent using Trizol RNA reagent (Thermo Fisher Scientific) and the PureLink RNA Mini Kit (Thermo Fisher Scientific). In brief, Trizol RNA reagents were added to the cells after medium removal to lyse the cells. After phase separation steps, the fraction containing RNA was applied to the column provided in the RNA Mini Kit to purify the total RNA according to the manual of the kit. Libraries were generated using TruSeq stranded RNA preparation kit (Illumina) and sequenced using Illumina HiSeq 2000 (single-end, 50 bp) in the High Throughput Genomic Sequencing Facility at UNC_Chapel Hill. Two independent RNA preparations for each cell line were sequenced.

Sequences were mapped to the mouse genome (mm10) using RNA STAR (Galaxy 2.6.0b-1). The numbers of mapped reads of each gene were counts using FeatureCounts (Galaxy 1.6.0.6). Data from FeatureCounts were further analyzed using Deseq2 (1.22.2) in the R program. Log2FoldChange (LFC) comparing to the CRY/PER/NR1D_KO cells was used for further analysis. The correlation analysis was performed using the “cor.test” function in the R program with the parameter “method = ‘Pearson’.” For visualization of the sequencing results, mapped reads were separated into two strands using Filter SAM or BAM, output SAM or BAM (Galaxy 1.8), converted to the bedgraph files using the Genome Coverage (Galaxy 2.27.0.0), and to the bigwig files using the Wig/BedGraph-to-bigWig (Galaxy 1.1.1). The raw sequencing data and the processed data were uploaded to the Gene Expression Omnibus (GSE157946).

### Dbp Expression Analysis of Serum- or Dexamethasone-Synchronized Cells

For serum synchronization, cells were seeded into 60 mm dishes the day before the experiment. Serum shock was performed by replacing the medium with the DMEM medium with 50% horse serum. After 2 h, the high serum medium was replaced with the DMEM medium with 10% FBS. Cells were collected every 2 h using TriReagent (Zymo Research) and were kept at −80°C. For dexamethasone treatment, cells were treated with 100 nM dexamethasone for 2 h. Then, the medium was changed to the DMEM medium with 10% FBS, and the cells were collected as serum shock experiments.

Total RNA was purified using the DirectZol RNA Miniprep kit (Zymo Research) following the manual. RNA concentration was determined by Qubit RNA HS kit (Thermo Fisher Scientific). The same amount of RNA was reverse-transcribed into cDNA using PrimeScript RT Reagent kit (Takara Bio). Real-time PCR analysis was performed using iQ SYBR Green Supermix and CFX96 Real-Time PCR Detection System (Biorad). The relative level of RNA to the untreated sample was calculated using the 2^∧^(−ΔΔCq) method using primary Gapdh RNA as the internal control. The primer sequences were provided in Supplementary Table.

### Dbp Expression Analysis of in Cry/Per/Nr1d_KO Cells Expressing CRY1-ER

The DNA fragment of the ligand-binding domain of the estrogen receptor (ER) was PCR-amplified from the pWZL-blast-PER2-ER (Ye et al., 2014). This fragment was inserted to the 3′-end of mouse Cry1 cDNA in a vector by restriction enzyme digestion and ligation. The CRY1-ER was PCR-amplified and was subcloned into the pWZL-blast by restriction enzyme digestion and ligation to make pWZL-blast-CRY1-ER. Cry/Per/Nr1d_KO cells were infected by the retrovirus carrying CRY1-ER fragment. After blasticidin selection (5 μg/ml), individual colonies were isolated, and the expression of CRY1-ER protein was checked by Western blot.

The Cry/Per/Nr1d_KO-CRY1-ER cells were maintained in the DMEM medium containing 10% FBS and 2.5 μg/ml blasticidin. The cells were seeded in the DMEM medium without blasticidin when performing experiments. The addition of 4-hydroxytamoxyfen (4-OHT) into the medium to 10 nM was used to induce CRY1-ER nuclear entry. RNAs were prepared at different time courses after 4-OHT treatment and were analyzed as mentioned above.

### Nuclear Fractionation and Western Blot

Antibody for detecting mouse CRY1 was provided by the laboratory of Aziz Sancar and was described previously (Ye et al., 2011). Antibodies for detection CLOCK (Bethyl Laboratories), BMAL1 (Bethyl Laboratories), CRY2 (Bethyl Laboratories), NR1D1 (Cell Signaling Technology), and NR1D2 (Santa Cruz Biotechnology) were from commercial sources.

For nuclear fractionation, cells were detached from the plate by trypsinization. After PBS wash, cells were lysed with hypotonic buffer (10 mM HEPES pH7.9, 1.5 mM MgCl_2_, 10 mM KCl, 0.3% Igepal-CA630). After centrifugation (400 g for 5 min), the supernatants were collected as cytosol fractions. The nuclear pellets were washed with hypotonic buffer and were lysed with NUN buffer (20 mM HEPES pH7.4, 300 mM NaCl, 1 M urea, and 10% glycerol). After centrifugation (15,000 g for 15 min), the supernatants were collected as the enriched nuclear fractions.

The protein concentrations of lysates were estimated by the QuantiChrom^TM^ Total Protein Assay Kit (BioAssay System). Equal amounts of total proteins were applied to the Western blot experiments.

The Western blot results were quantified by the Image Lab software (Bio-Rad Laboratories). For estimating the relative levels of CRY1-CLOCK-BMAL1 in the nucleus, CRY1 levels in the nuclear fractions were first normalized to the BMAL1 levels and then compared to the group without 4-OHT treatment.

## Results

### Generation of Mouse Embryonic Fibroblast Cell Line Lacking CRY1, CRY2, PER1, PER2, NR1D1, and NR1D2 (CPN_KO Cells)

**FIGURE 1:**
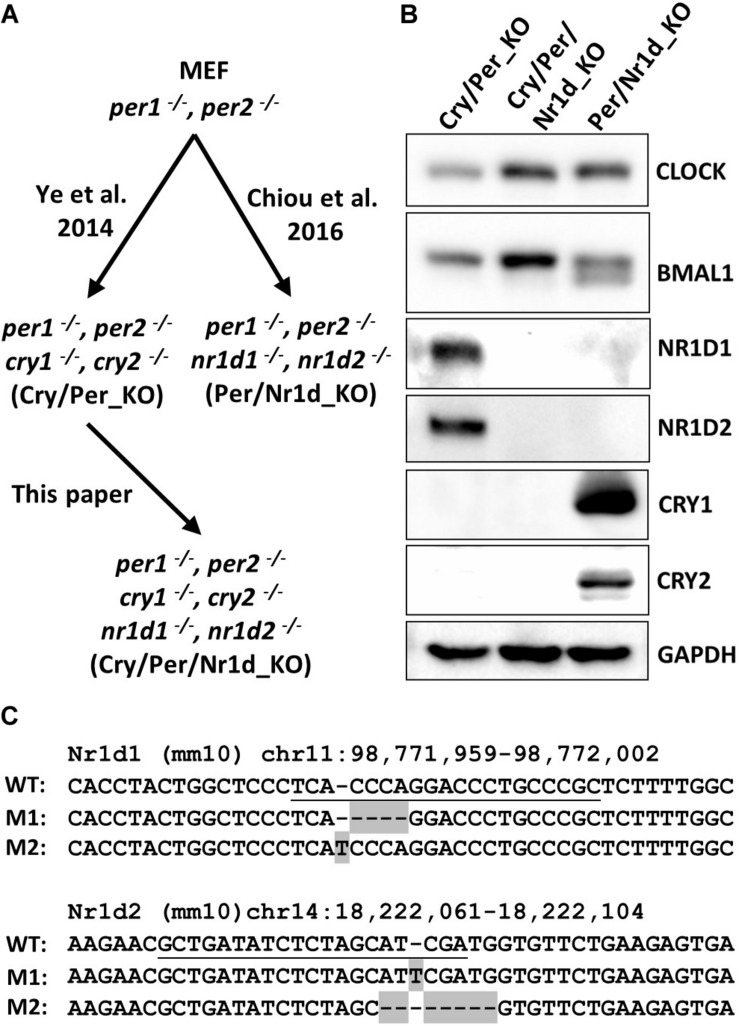
Characterization of Cry/Per/Nr1d_KO cells. **(A)** The relations of Cry/Per_KO, Cry/Per/Nr1d_KO, and Per/Nr1d_KO cells were shown. **(B)** Western blot analysis of CLOCK, BMAL1, NR1D1, NR1D2, CRY1, and CRY2 protein in these three cell lines were shown. The GAPDH was detected as the loading control. **(C)** The wild-type (WT) sequences (mouse genome: mm10) of Nr1d1 and Nr1d2 around the CRISPR/Cas9 targeting sites and the genome sequences from Cry/Per/Nr1d_KO cells were aligned. The guide RNA targeting sequences were underlined. The mutated sequences in Cry/Per/Nr1d_KO cells were shaded.

To study the mechanism of TTFL without complicated interference between CRY, PER, and NR1D proteins, we attempted to generate a cell line lacking CRY1, CRY2, PER1, PER2, NR1D1, and NR1D2 (Cry/Per/Nr1d_KO). This cell line would be an advanced “*in cellulo*” biochemical system to study the function of proteins in the TTFL. We selected the MEF cell line lacking CRY1, CRY2, PER1, and PER2 (Cry/Per_KO) (Ye et al., 2014) as the parental cells. The Nr1d1 and Nr1d2 genes of the parental cells were mutated using the CRISPR/Cas9 system. Lentivirus expressing Cas9 protein and the guide RNA sequences targeting Nr1d1 and Nr1d2 was prepared as the previously published method for the generation of MEF cells lacking PER1, PER2, NR1D1, and NR1D2 (Per/Nr1d_KO) (Chiou et al., 2016). Figure 1A showed the relations of Cry/Per_KO, Per/Nr1d_KO, and Cry/Per/Nr1d_KO cells. We successfully isolated one clone lacking detectable NR1D1 and NR1D2 proteins from 12 clones in the first screening. We further compared the protein levels of CRY1, CRY2, NR1D1, NR1D2, CLOCK, and BMAL1 in the Cry/Per/Nr1d_KO cells with Cry/Per_KO and Per/Nr1d_KO cells (Figure 1B). Comparing to Cry/Per_KO cells, NR1D1 and NR1D2 proteins in Cry/Per/Nr1d_KO cells were undetectable as published Per/Nr1d_KO cells. Associated with the lack of NR1D proteins, CLOCK and BMAL1 proteins were higher in the Cry/Per/Nr1d_KO cells. Transcription of Bmal1 was repressed by NR1D proteins (Preitner et al., 2002), and thus, more BMAL1 proteins in Cry/Per/Nr1d_KO cells than in Cry/Per_KO cells was due to the increase of Bmal1 transcription in the absence of NR1D proteins. Cry/Per/Nr1d_KO cells did not express CRY1 and CRY2 proteins as the parental Cry/Per_KO cells. It has been shown that BMAL1 protein was hyperphosphorylated in Cry-deficient cells (Tamaru et al., 2003). We also observed more hyperphosphorylated BMAL1 in the Cry/Per/Nr1d_KO and the Cry/Per_KO cells than in the Per/Nr1d_KO cells expressing endogenous CRY1 and CRY2 proteins.

We also analyzed the genomic DNA sequences around the CRISPR/Cas9-targeting sites of Nr1d1 and Nr1d2 genes of the Cry/Per/Nr1d_KO cells. We got two types of Nr1d1 sequences from Cry/Per/Nr1d_KO cells with 4 bp deletion and 1 bp insertion comparing to the wild type. For Nr1d2 genes, we also got two types of sequences with 1 bp insertion and 7 bp deletion (Figure 1C). These data suggested that both alleles of Nr1d1 and Nr1d2 genes were frameshift-mutated in the Cry/Per/Nr1d_KO cells.

### Transcriptome Analysis of Cry/Per_KO, Per/Nr1d_KO, and Cry/Per/Nr1d_KO Cells

In the TTFL model, CRY could inhibit CLOCK-BMAL1 through direct interaction on DNA, and NR1D could inhibit CLOCK-BMAL1 by decreasing the Bmal1 level. In another way, CRY could increase the BMAL1 level through inhibiting Nr1d transcription, and NR1D could activate CLOCK-BMAL1 through decreasing CRY level. However, the rhythmic Bmal1 transcription is dispensable for the intracellular circadian rhythm (Liu et al., 2008; Xu et al., 2015). To distinguish the effect of BMAL1 level change by NR1D and the effect of BMAL1 activity by CRY on the transcriptome, we analyzed the transcriptomes of Cry/Per_KO, Per/Nr1d_KO, and Cry/Per/Nr1d_KO cells by RNA-sequencing (RNA-seq). The difference of transcriptome between Cry/Per/Nr1d_KO and Cry/Per_KO cells could be explained by the expression of endogenous NR1D1 and NR1D2 proteins regardless of the expression of CRY and PER protein. Also, CRY-mediated regulation could be analyzed by comparing the transcriptome of Per/Nr1d_KO and Cry/Per/Nr1d_KO cells.

**FIGURE 2:**
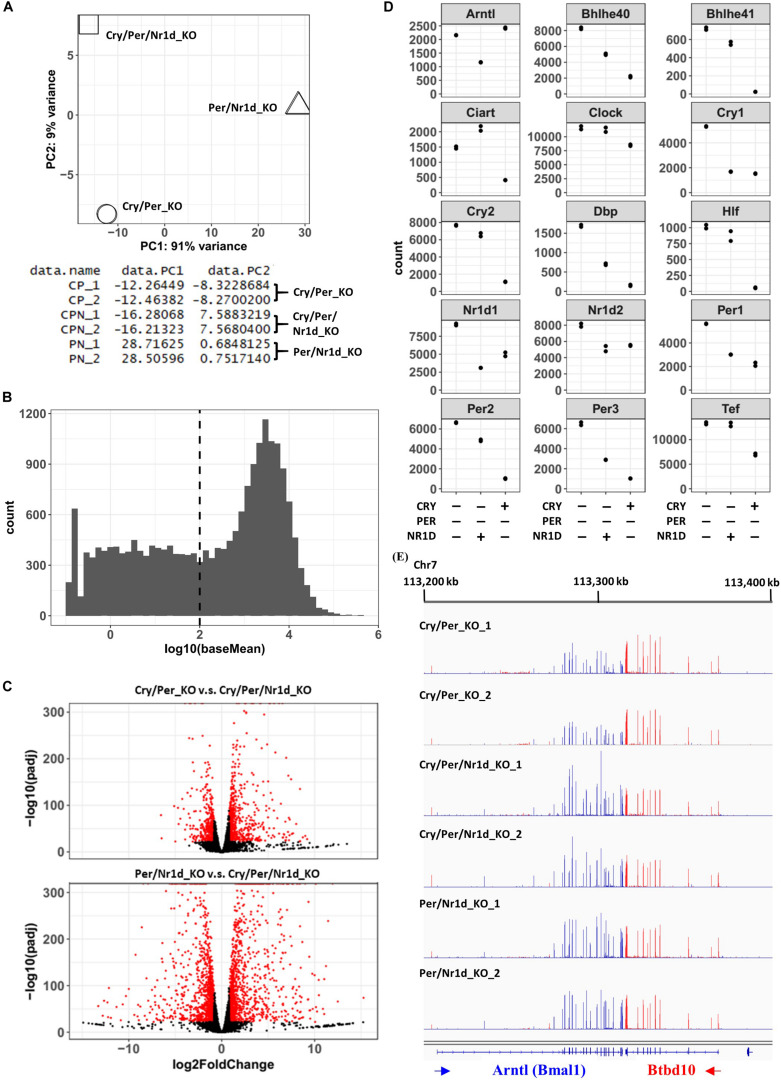
RNA-Seq Analysis of Cry/Per_KO, Per/Nr1d_KO, and Cry/Per/Nr1d_KO Cells. **(A)** The principal component analysis was performed using Deseq2, and the data were shown and was plotted using R software. **(B)** The histogram showed the distribution of baseMean calculated by Deseq2. The dashed line indicated the position that baseMean was equal to 100. **(C)** Volcano blots of Cry/Per_KO cells vs. Cry/Per/Nr1d_KO cells and Per/Nr1d_KO cells vs. Cry/Per/Nr1d_KO cells were shown. Genes with significant differences were colored in red. **(D)** The read counts of different genes calculated by Deseq2 were extracted and were plotted. Each spot represented the value of each experiment. **(E)** The figure was the screenshot of RNA-seq data visualized by the IGV genome browser. RNA-seq datamapped to different strands of the genome were displayed in different colors, and then were overlayed to display. For better visualization quality, sample names, gene names, and the locations of the genome were added manually.

The association between RNA-seq data from two independent experiments of each cell line was analyzed by the principal component analysis (Figure 2A). We filtered out the genes with lower expression levels (baseMean < 100) according to the distributions of baseMean of all genes (Figure 2B), and compared the expression 11,912 genes in these three cell lines. The criteria we used for selecting the changed genes were described as follows: (1) the level of change was more than 75% [the absolute value of log2FoldChange > log2(1.75)], and (2) the adjusted *p*-value was less than 1e-22. Comparing with Cry/Per/Nr1d_KO cells, 1,090 genes expressed differently in Cry/Per_KO cells, and 2,527 genes expressed differently in Per/Nr1d_KO cells (Figure 2C). From RNA-seq data, we selected circadian genes, including Clock, Arntl (Bmal1), Cry1, Cry2, Per1, Per2, Per3, Nr1d1, Nr1d2, Dbp, Hlf, Tef, Bhlh40, Bhlh41, and Ciart to compare the effect of CRY and NR1D on CLOCK-BMAL1 and its target genes (Figure 2D). Comparing to Cry/Per/Nr1d_KO cells, Per/Nr1d_KO cells (with CRY1 and CRY2 proteins) showed decreased RNA levels of Cry1, Cry2, Per1, Per2, Per3, Nr1d1, Nr1d2, Dbp, Hlf, Tef, Bhlh40, and Bhlh41. These genes were known CLOCK-BMAL1-regulated genes, and thus the data suggested that CRY proteins repressed CLOCK-BMAL1-mediated transcription in the absence of PER and NR1D proteins. In Per/Nr1d_KO cells, Clock mRNA was slightly lower than, and Arntl mRNA was similar to the level in Cry/Per/Nr1d_KO cells. These data indicated that CRY could not affect Clock and Bmal1 mRNA levels in the absence of NR1D proteins. In Cry/Per_KO cells (with NR1D1 and NR1D2 proteins), Arntl and Cry1 mRNAs were lower than those in the Cry/Per/Nr1d_KO cells as expected, because known RRE element was identified on these two genes. However, the influences of NR1D proteins to other CLOCK-BMAL1-regulated genes were gene-specific. The mRNA levels of Nr1d1, Nr1d2, Per1, and Per3 in Cry/Per_KO cells were lower than Cry/Per/Nr1d_KO cells as the levels in Per/Nr1d_KO cells. However, the mRNA levels of Per2, Bhlh40, and Dbp in Cry/Per_KO cells were lower than Cry/Per/Nr1d_KO but were higher than Per/Nr1d_KO cells. Furthermore, the mRNA levels of Cry2, Hlf, and Tef in Cry/Per_KO cells were similar to the levels in Cry/Per/Nr1d_KO cells. RNA-seq data visualized by genome browser also presented the different expression levels of Arntl genes between Cry/Per/Nr1d_KO and Cry/Per_KO cells, but not between Cry/Per/Nr1d_KO_and Per/Nr1d_KO cells (Figure 2E).

**FIGURE 3:**
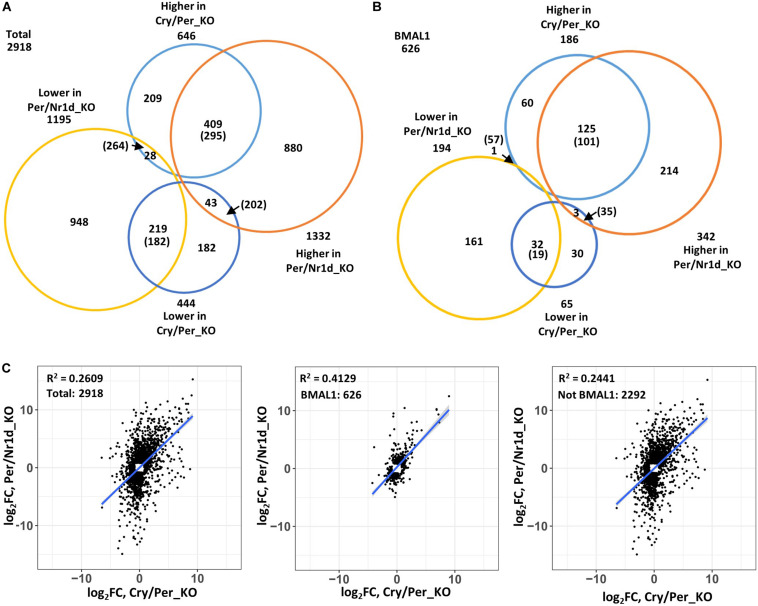
Correlation analysis of NR1D and CRY affected genes. **(A)** Genes expressing differently to Cry/Per/Nr1d_KO cells were subgrouped based on their changes in Per/Nr1d_KO cells and Cry/Per_KO cells. The numbers indicated the numbers of genes in each group. The numbers in the parentheses were the mean values from random sampling for 100 times. Genes within the list of putative CLOCK-BMAL1-regulated genes were selected and were shown in **(B)**. **(C)** The expressional changes of individual genes in Per/Nr1d_KO (*y*-axis) and Cry/Per_KO (*x*-axis) cells were plotted. The correlation was analyzed using the linear regression model (“lm”). The R-squared values and the regression lines were shown in the figures. Data from total selected genes were plotted in the left panel or were subgrouped into BMAL1-regulated (the middle panel) or not BMAL1-regulated (the right panel).

To compare genes affected by the loss of CRY proteins (Per/Nr1d_KO v.s. Cry/Per/Nr1d_KO) or NR1D proteins (Cry/Per_KO v.s. Cry/Per/Nr1d_KO), we focused on the 2,918 genes that expressing differently between Cry/Per_KO and Cry/Per/Nr1d_KO (*n* = 1,090) or between Per/Nr1d_KO and Cry/Per/Nr1d_KO (*n* = 2,527) cells. Among these genes, 628 genes were affected by both CRY and NR1D in the same trend (higher or lower in both Cry/Per_KO and Per/Nr1d_KO comparing to Cry/Per/Nr1d_KO cells). This number was 57.6% of the number of NR1D affected genes and 24.1% of the number of CRY affected genes). The number of genes contrarily affected by CRY and NR1D was 71 (6.5% of NR1D affected genes, 2.8% of CRY affected genes). Others were uniquely affected by NR1D or CRY (35.9% of NR1D affected genes, 73.1% of CRY affected genes) (Figure 3A). We did a random sampling test (*n* = 100) to check whether these numbers were closed to random events. We randomly selected 1,195 (low group) and 1,332 (high group) genes as the number of genes expressing lower or higher in Per/Nr1d_KO cells and selected 444 (low group) and 646 (high group) genes as the number of genes expressing lower or higher in Cry/Per_KO cells. The mean values of the number of overlapping genes in the same group and the opposite group were 477 and 466. These data suggested that the overlapping of CRY and NR1D affected genes was not just a random event.

Because both NR1D and CRY could affect CLOCK-BMAL1-regulated genes, we selected putative CLOCK-BMAL1-regulated genes based on previously published BMAL1-chromatin immunoprecipitation sequencing data from the same Per/Nr1d_KO background (Chiou et al., 2016). Total 626 CLOCK-BMAL1-regulated genes expressing differently between Cry/Per_KO and Cry/Per/Nr1d_KO (*n* = 251, 40.1%) or between Per/Nr1d_KO and Cry/Per/Nr1d_KO (*n* = 536, 85.6%) cells. Among these genes, 157 genes were affected by both CRY and NR1D knockout in the same trend (62.5% of NR1D affected CLOCK-BMAL1-regulated genes, 29.3% of CRY affected CLOCK-BMAL1regulated genes). This number was higher than the mean value (120) from the random sampling test. Only four genes (the mean value from random sampling test was 92) were oppositely affected by CRY and NR1D (1.6% of NR1D affected CLOCK-BMAL1-regulated genes, 0.7% of CRY affected CLOCK-BMAL1-regulated genes). The numbers of CLOCK-BMAL1-regulated genes uniquely affected by NR1D or CRY were 90 (35.8% of NR1D-regulated) and 375 (59.9% of CRY-regulated) (Figure 3B).

We further analyzed the correlation of the CRY or NR1D-affected genes (Figure 3C). The expression of these genes showed a positive correlation (Pearson’s correlation coefficient = 0.5108, *p*-value < 2.2e-16) between Cry/Per_KO and Per/Nr1d_KO cells. The correlation was increased when putative BMAL1-regulated genes were selected (Pearson’s correlation coefficient = 0.6426, *p*-value < 2.2e-16). Reversely, the correlation was decreased when putative BMAL1-regulated genes were excluded (Pearson’s correlation coefficient = 0.4940, *p*-value < 2.2e-16). Our RNA-seq data suggested that CRY and NR1D could regulate CLOCK-BMAL1-regulated genes independently. In another way, many genes might be regulated by CRY or NR1D in a CLOCK-BMAL1-independent manner.

### Responses of Dbp and Ciart Transcription in Cry/Per_KO, Per/Nr1d_KO, and Cry/Per/Nr1d_KO Cells After Serum Shock and Dexamethasone Shock

Synchronization of cells by serum shock or dexamethasone has been used for the study of the circadian clock in the cellular model (Balsalobre et al., 1998, 2000a). However, how these treatments affected core TTFL was unclear. We have three cell lines expressing different proteins in the TTFL from the same source. Thus, we analyzed the activity of CLOCK-BMAL1 in these three different cell lines after serum shock or dexamethasone shock to find out which protein in the TTFL responded to the synchronization signals. Neither Per/Nr1d_KO, Cry/Per_KO, nor Cry/Per/Nr1d_KO cells could generate a stable oscillation of clock-controlled genes after synchronization because of the loss of complete TTFL. We focused on a shorter time course (<8 h) after the release of synchronization to analyze the direct effects of treatment on TTFL. We analyzed the primary transcript levels of two CLOCK-BMAL1 target genes, Dbp and Ciart, by RT-qPCR to represent the transcription of these genes. The primary transcript of Gapdh was selected as the internal control for possible global effect on global transcription.

**FIGURE 4:**
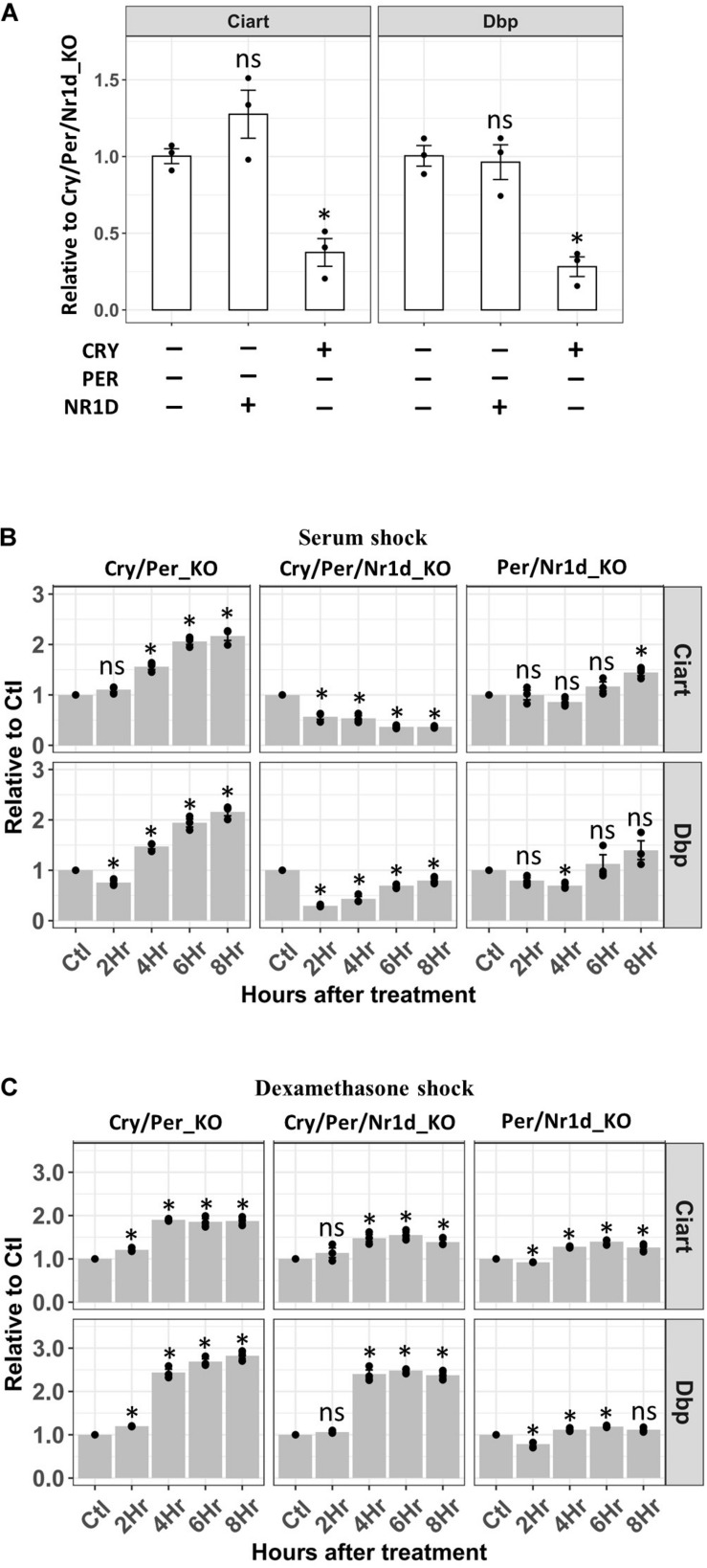
RT-qPCR analysis of the Dbp and Ciart primary mRNA after serum or dexamethasone shock. **(A)** RT-qPCR analyzed primary mRNA levels of Ciart and Dbp in Cry/Per/Nr1d_KO, Cry/Per_KO, and Per/Nr1d_KO cellswere shown. Data were first normalized to the primary mRNA of Gapdh, then were normalized to the average in Cry/Per/Nr1d_KO cells (*y*-axis). Data were shown as mean with standard error. Data from independent experiments (point, *n* = 3) were also shown in the plot. Statistical analysis was performed using Student’s *t*-test comparing to the Cry/Per/Nr1d_KO cells. *P*-values < 0.05 were considered significant. Asterisk denoted the significant difference. Data without significant difference were labeled with “ns.” **(B)** RT-qPCR analyzed primary mRNA levels of Ciart and Dbp in Cry/Per/Nr1d_KO, Cry/Per_KO, and Per/Nr1d_KO cells after serum shock were shown. Data were first normalized to the primary mRNA of Gapdh, then were normalized to the values from the same cells without treatment (*y*-axis). Cells without treatment, 2, 4, 6, 8 h after treatments were analyzed (*x*-axis). Statistical analysis was performed using Student’s *t*-test comparing to the untreated control of the same cells. *P*-values < 0.05 were considered significant. Asterisk (*) denoted the significant difference. Data without significant difference were labeled with “ns.” **(C)** RT-qPCR analyzed primary mRNA levels of Ciart and Dbp in Cry/Per/Nr1d_KO, Cry/Per_KO, and Per/Nr1d_KO cells after dexamethasone shock were shown. Statistical analysis was performed as in **(B)**.

Transcription of Ciart and Dbp were lower in the Per/Nr1d_KO cells expressing endogenous CRY proteins than the other two cells without treatment. However, the expression of NR1D proteins in Cry/Per_KO cells did not affect the transcription of Ciart and Dbp comparing to Cry/Per/Nr1d_KO cells (Figure 4A). In Cry/Per/Nr1d_KO cells, serum shock decreased the primary mRNA of Dbp and then gradually recovered. The primary RNA levels of Ciart gradually decreased after serum shock. These suggested that serum shock inhibited the activity of CLOCK-BMAL1 in the absence of CRY, PER, or NR1D proteins (Figure 4B, middle). In the presence of NR1D proteins (Cry/Per_KO cells), serum shock did not inhibit the transcription of Dbp or Ciart. Instead, transcription of these genes was induced by serum shock with a time delay (Figure 4B, left). In Per/Nr1d_KO cells, we did not observe apparent effects on the transcription of these two genes after serum shock (Figure 4B, right). Based on these data, we proposed that serum shock directly inhibits the activity of CLCOK-BMAL1 to decrease the transcription of target genes. For cells with high NR1D levels, decreased NR1D by decreasing CLOCK-BMAL1 activity induced the transcription of Bmal1. Increased CLOCK-BMAL1 compensated the decreased activity and caused the increase of Dbp and Ciart transcription at later time points. For cells with high CRY levels, the transcriptions of Dbp and Ciart were maintained at the lower levels (Figure 4A). Thus, serum shock did not cause apparent effects on Per/Nr1d_KO cells.

Horse serum contains many substances, including glucocorticoids. Dexamethasone is an agonist of glucocorticoids to activate the glucocorticoid receptor. Thus, we wanted to compare the response of dexamethasone shock to the serum shock in these three cell lines. Dexamethasone did not repress the transcription of Dbp or Ciart. Instead, we could detect the increase of Dbp transcription four hours after the release of dexamethasone in Cry/Per_KO and Cry/Per/Nr1d_KO cells (Figure 4B, left and middle) but not in Per/Nr1d_KO cells (Figure 4B, right). The effects of dexamethasone on Ciart could be observed in Cry/Per_KO cells and was weaker than the effects on Dbp. Based on our data, we concluded that the mechanisms of how serum and dexamethasone affect TTFL are different.

### Study CRY1-Mediated Transcriptional Inhibition of Dbp and Ciart in Cry/Per/Nr1d_KO Cells

**FIGURE 5:**
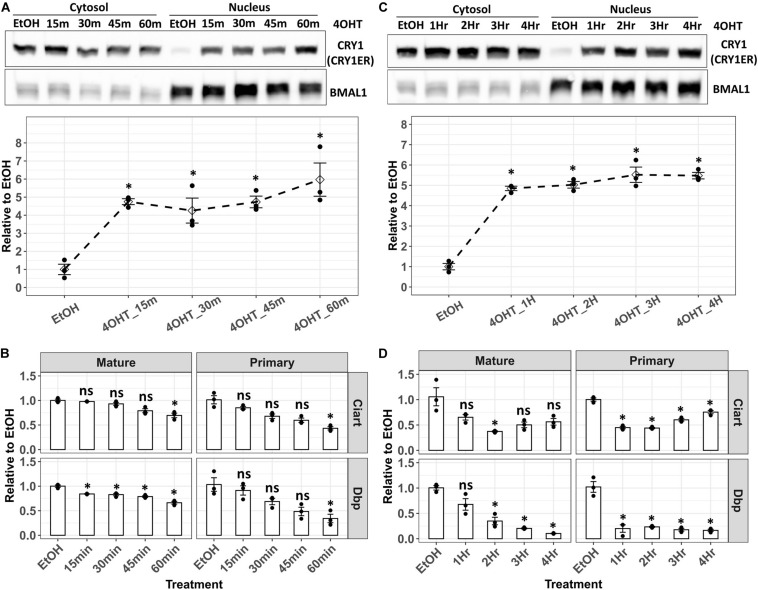
Analysis of the Dbp and Ciart primary and mature mRNA in Cry/Per/Nr1d_KO-CRY1-ER cells after 4-OHT treatment**. (A)** Cry/Per/Nr1d_KO-CRY1-ER cells were treated with 4-OHT for 15–60 min. After the fractionation, CRY1-ER proteins were analyzed by Western blots. BMAL1 was used as the control for the nuclear fractionation and represented the total levels of CLOCK-BMAL1 (with and without CRY) in the nucleus. The lower panel was the relative levels of CRY1-ER in the nucleus to the cells treated with the vehicle (EtOH, 60 min). Data were first normalized to the BMAL1 levels, then were normalized to the average of vehicle control. The diamonds were the mean of three independent experiments. Error bars were the standard errors, and the points were the individual data. Statistical analysis was performed using Student’s *t*-test comparing to the vehicle control (EtOH). *P* < 0.05 were considered significant. Asterisk (*) denoted the significant difference. **(B)** Cells were treated with 4-OHT as in **(A)**. RT-qPCR analyzed primary and mature mRNA levels were shown. Data were first normalized to the primary or mature mRNA of Gapdh, then were normalized to the average of vehicle control (y-axis). Data were shown as mean with standard error. Data from independent experiments (point, *n* = 3) were also shown in the plot. Statistical analysis was performed using Student’s *t*-test comparing to the vehicle control (EtOH). *P*-values < 0.05 were considered significant. Asterisk (*) denoted the significant difference. Data without significant difference were labeled with “ns.” **(C)** Cry/Per/Nr1d_KO-CRY1-ER cells were treated with 4-OHT for 1–4 h. Cells treated with EtOH for 4 h were the controls. Experiments and data analysis were the same as **(A)**. **(D)** The treatments of cells were identical to **(C)**, and the experiments and data analysis were the same as **(B)**.

Lack of CRY1, CRY2, PER1, PER2, NR1D1, and NR1D2 proteins in Cry/Per/Nr1d_KO cells benefited the study of each of them individually. We combined a previously published tamoxifen-controlled nuclear entry system (Ye et al., 2014) and this unique cell line to study the transcriptional repression of Dbp by CRY1. Theoretically, if we could control the nuclear CRY1 level rhythmically, Dbp transcription would be rhythmic, too. We established Cry/Per/Nr1d_KO-CRY1-ER cells expressing CRY1 fused with an estrogen receptor ligand-binding domain. The addition of 4-hydroxytamoxifen (4-OHT) released CRY1-ER from the cytosol to the nucleus through binding to the estrogen receptor ligand-binding domain. The nuclear levels of CRY1-ER increased within 15 min after the addition of 4-OHT (Figures 5A,C). We could not observe the correlation between nuclear CRY1-ER and the treatment time (Figures 5A,B). Thus, we thought the nuclear level of CRY1-ER reached a saturated status within 15 min after adding 4-OHT. However, the effects of CRY1 on transcriptional inhibition of Dbp and Ciart showed a time-dependent manner (Figures 5B,D). The decrease of Dbp and Ciart primary mRNA could be observed within an hour (Figure 5B, right panel), and the decrease of Dbp and Ciart mature mRNA needed several hours (Figure 5D, left panel). We observed that the primary mRNA levels of Ciart, but not Dbp, were gradually recovered after 3 h of treatment (Figure 5D, right panel), representing that another regulatory mechanism independently activated the transcription of Ciart.

**FIGURE 6:**
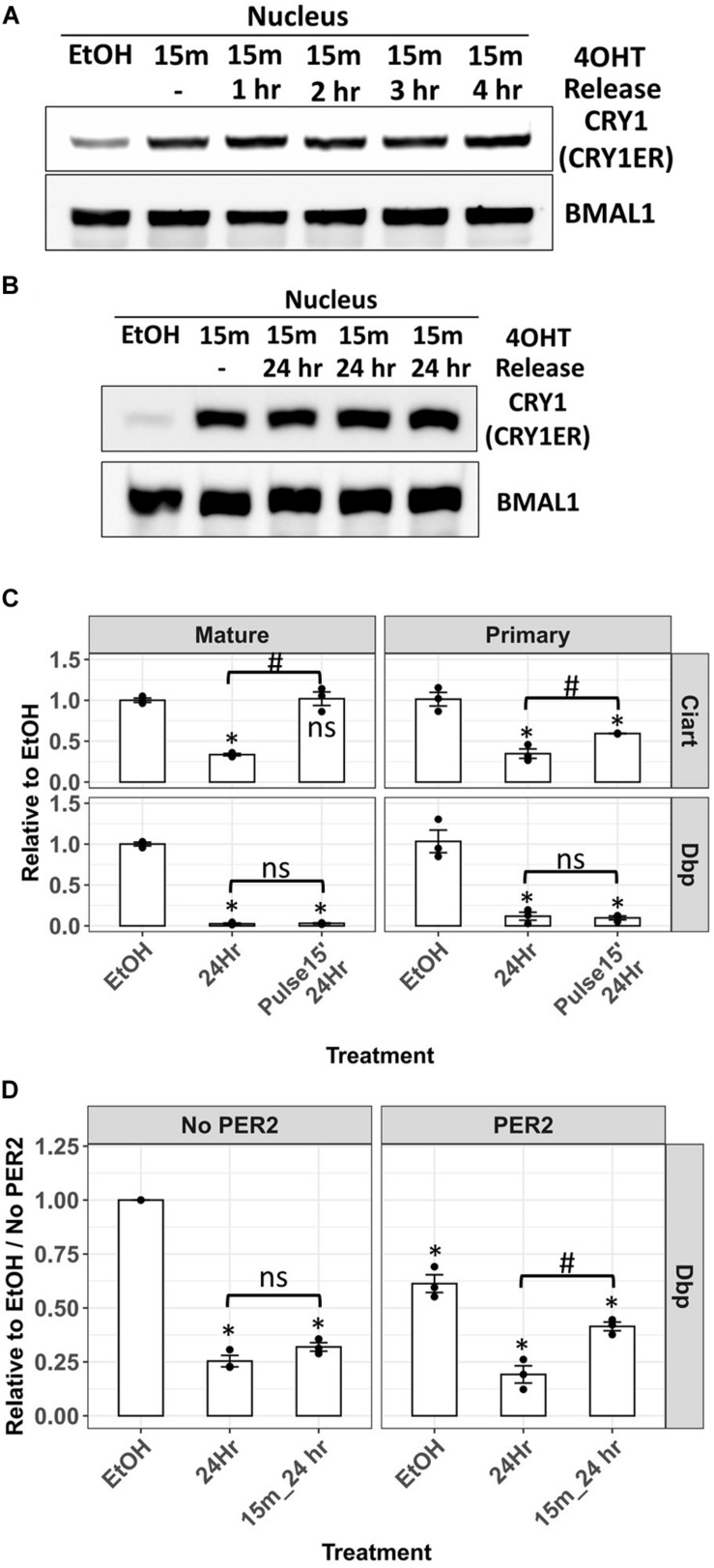
Analysis of the Dbp primary mRNA in Cry/Per/Nr1d_KO-CRY1-ER cells after 4-OHT treatment and release. Nuclear levels of CRY1-ER in Cry/Per/Nr1d_KO-CRY1-ER cells treated with 4-OHT for 15 min and released for 1–4 h **(A)** or 24 h **(B)** were analyzed by Western blot after fractionation. BMAL1 was used as the loading control. **(C)** RT-qPCR analysis of primary and mature mRNA levels of Ciart and Dbp from cells treated with vehicle for 24 h (EtOH), 4-OHT for 24 h (24 h), and 4-OHT for 15 min and released for 24 h (pulse 15’, 24 h) were shown. Statistical analysis was performed using Student’s *t*-test. *P*-values < 0.05 were considered significant. Asterisk denoted the significant difference to the vehicle control. Hashtag denoted the significant difference between the 24 h treatment of 4-OHT and the 24 h release after 15 min 4-OHT treatment. Data without significant difference were labeled with “ns.” **(D)** Cells were transfected with the control plasmid (pcDNA4-Myc-His) or with the plasmid expressing PER2 (pcDNA3-PER2-V5-His) 24 h before 4-OHT treatment. The 4-OHT treatment, RT-qPCR, and data analysis were the same as **(C)**. Statistical analysis was performed using Student’s *t*-test. *P* < 0.05 were considered significant. Asterisk (*) denoted the significant difference to the vehicle control without PER2 expression. Hashtag (#) denoted the significant difference between the 24 h treatment of 4-OHT and the 24 h release after 15 min 4-OHT treatment. Data without significant difference were labeled with “ns.”

Next, we analyzed the nuclear distribution of CRY1-ER after the removal of 4-OHT. The cells were treated with 4-OHT for 15 min, and then the medium was changed after PBS wash. Unexpectedly, nuclear CRY1-ER was unchanged after the removal of 4-OHT for 4 h (Figure 6A). We extended the release time to 24 h and found that nuclear CRY1-ER after removing 4-OHT for 24 h was similar to the level just after 15 min 4-OHT treatment (Figure 6B). In the analysis of Dbp and Ciart mRNA, Dbp primary and mature mRNA after 24 h of the release of 4-OHT were as low as the levels without release (Figure 6C, lower panel). The results were consistent with the nuclear levels of CRY1-ER and suggested that CRY1-mediated transcriptional repression of Dbp persisted for 24 h after 4-OHT removal. The primary mRNA of Ciart was partially recovered, and the mature mRNA was fully recovered after 24 h release of 4-OHT (Figure 6C, upper panel). However, this may be through a TTFL-independent mechanism as the observation in Figure 5D.

In our previous studies, PER2 could remove CLOCK-BMAL1 from the promoter of Dbp genes and repress the transcription of Dbp (Ye et al., 2014). In another way, PER2 could also remove CLOCK-BMAL1-CRY to derepress the transcription of downstream genes, like Cry1 (Chiou et al., 2016). To understand whether PER is required for the reactivation of Dbp transcription repressed by CRY, we transiently transfected the plasmid expressing PER2 protein 24 h before 4-OHT treatment. Without 4-OHT treatment, Dbp primary mRNA in the cells expressing PER2 was lower than the cells without PER2. These data could be explained by the removal of CLOCK-BMAL1 from the promoter. The expression of PER2 did not affect the repression of Dbp by 4-OHT-induced CRY1 nuclear entry. Nevertheless, the transcriptional recovery of Dbp after 4-OHT removal could only be observed in the cells expressing PER2 (Figure 6D). Our data suggested that PER is required for the release of CRY-mediated transcriptional repression.

## Discussion

The transcription-translation feedback loop was the core model of the circadian clock. However, studying the detailed mechanism was complicated. Experiments using purified proteins could not link to the transcription in the absence of an *in vitro* transcription system using endogenous promoter. Animals were suitable for the study of circadian regulation of physiological functions, but the detailed mechanism was hard to elucidate due to the communications between organs and tissues. Cells had a self-sustained circadian clock in the cultured condition that played a suitable model to study the mechanism. Genetic deficient mouse embryonic fibroblast cells had been used for many circadian studies. We generated a new cell line, called Cry/Per/Nr1d_KO cells, lacking CRY, PER, and NR1D proteins by CRISPR/Cas9 approach and analyzed the transcriptome of Cry/Per/Nr1d_KO, Per/Nr1d_KO, and Cry/Per_KO cells by RNA-sequencing. Using Dbp and Ciart as the readout of CLOCK-BMAL1 activity, we found that serum and dexamethasone treatment affected TTFL through different mechanisms. We found that serum, but not dexamethasone, inhibited CLOCK-BMAL1 in the absence of CRY, PER, and NR1D proteins. We studied CRY1-mediated transcriptional repression of Dbp in Cry/Per/Nr1d_KO cells and found that PER is required for the release of CRY-mediated transcriptional repression.

Cry/Per/Nr1d_KO cells provided a new tool to study the TTFL of the circadian clock biochemically. Researchers could study the regulation of CLOCK-BMAL1 and the functions of CRY, PER, and NR1D separately without the influence of feedback in Cry/Per/Nr1d_KO cells. For example, analysis of wild-type and mutant CRY1 in Cry/Per/Nr1d_KO cells helps elucidate the difference in the repression of CLOCK-BMAL1. Changes of PER activity or the CLOCK-BMAL1 quantity would not be the issue in Cry/Per/Nr1d_KO cells. However, from the biological view, Cry/Per/Nr1d_KO cells was a very extreme condition which may not exist in the physiological situation. We thought that Cry/Per/Nr1d_KO cells might mimic a situation that CLOCK-BMAL1 activity was reached the peak before the accumulation of CRY, PER, and NR1D proteins. Different situations could be mimicked by expressing different combinations of CRY, PER and NR1D proteins.

From the transcriptome analysis, we found 2,517 genes expressed differently between Cry/Per/Nr1d_KO and Per/Nr1d_KO cells. This number was around threefold of the number of genes expressed differently between Cry/Per/Nr1d_KO and Cry/Per_KO cells (790 genes). From the data of selected CLOCK-BMAL1-regulated genes, CRY proteins showed more robust represser activities than NR1D proteins. A more potent transcriptional repressor might cause more dramatic effects on the transcriptome. We also found that the overlapping of putative CLOCK-BMAL1-regulated genes and differential expressed genes was low. Only 626 genes (21% of affected genes) closed to the BMAL1 binding site identified from a similar condition (Chiou et al., 2016). Besides CLOCK-BMAL1, CRY proteins could regulate other transcription factors. In mouse liver, CRY1 or CRY2 could bind to many positions of the genome without CLOCK-BMAL1 binding (Koike et al., 2012). Although NR1D proteins regulate the levels of CLOCK-BMAL1, genes with the RRE element would be the targets of NR1D proteins. Besides directly affected by CRY or NR1D, genes might be affected by the transcription factors or other proteins controlled by CRY or NR1D. From another viewpoint, many putative CLOCK-BMAL1 regulated genes were not affected by either CRY or NR1D. Among 3,261 putative genes, 536 genes were affected by CRY, and 251 genes were affected by NR1D. In our previous study, PER2 removed CLOCK-BMAL1 globally, but only small proportions of genes were affected by the increase of PER2 in the nucleus (Chiou et al., 2016). Thus, CLOCK-BMAL1 activity was not required for the transcription of many regulated genes. Their transcriptional levels depended more on other factors than on CLOCK-BMAL1. CLOCK-BMAL1 played another layer of regulation for the oscillation. The effect of CLOCK-BMAL1 was masked in the absence of synchronization.

Synchronization was the essential step to observe the oscillation of circadian genes. Different treatments, including serum shock (Balsalobre et al., 1998), dexamethasone (Balsalobre et al., 2000a), and forskolin (Balsalobre et al., 2000b), had been reported and been used for this purpose. We tested the response of Dbp and Ciart transcription after serum or dexamethasone treatment in cells with different circadian proteins to elucidate the mechanism of synchronization. Glucocorticoid response element (GRE) had been identified in the promoter of Per2 genes (Cheon et al., 2013). Dexamethasone might synchronize the cells by inducing the expression of Per2. The induction of Per2 by dexamethasone would inhibit the transcription of Dbp. In our system lacking PER2 protein, we could not detect the decrease of Dbp transcription after dexamethasone treatment. Instead, increased Dbp transcription could be observed 4 h after treatment in Cry/Per_KO or Cry/Per/Nr1d_KO cells (Figure 4C). Delayed activation of Dbp might be the secondary effect through other genes regulated by glucocorticoid signals. Serum shock inhibited the transcription of Dbp in the cells lacking CRY, PER, and NR1D proteins (Cry/Per/Nr1d_KO cells). These data suggested that CLOCK-BMAL1 activity could be directly inhibited by serum. However, in cells expressing NR1D proteins (Cry/Per_KO cells), the inhibition of Dbp transcription was compensated (Figure 4B). Through literature searching, we proposed the following hypothesis to explain the observation in our system. Recently, Tip60 protein was identified as the acetyltransferase of Bmal1. Tip60 protein catalyzed the K538 acetylation of BMAL1 and activated BMAL1 (Petkau et al., 2019). Tip60 protein could be phosphorylated on S86 by GSK3b to activate its acetyltransferase activity (Lin et al., 2012). Besides, serum shock induced the phosphorylation of GSK3b on S9 to inhibit the kinase activity (Yin et al., 2006). Thus, serum shock inhibited the CLOCK-BMAL1 activity probably through decreasing the activity of GSK3b and Tip60. However, NR1D1 was also the target of GSK3b. Decreased GSK3b activity by serum shock caused the decrease of NR1D1 protein and thus increased Bmal1 transcription (Yin et al., 2006), explaining why Dbp transcription in the Cry/Per_KO cells after serum shock was not decreased but was increased at later time points.

In the canonical TTFL model, transcription of CLOCK-BMAL1-regulated genes could be downregulated by CRY, PER, or NR1D proteins. Dbp transcription in the Per/Nr1d_KO cells was lower than in the Cry/Per/Nr1d_KO cells suggesting that the repressor activity of CRY is PER-independent as previously published (Ye et al., 2014). Neither serum-induced Dbp repression nor dexamethasone-induced Dbp activation in the Cry/Per/Nr1d_KO cells was observed in the Per/Nr1d_KO cells. Although CRY proteins negatively regulated their transcription, endogenous CRY1 and CRY2 in the Per/Nr1d_KO cells were sufficient to repress the Dbp transcription. CRY represses transcription through forming CLOCK-BMAL1-CRY-DNA complex. PER can remove CLOCK-BMAL1-CRY from DNA. In the absence of PER, the CLOCK-BMAL1-CRY-DNA complex might be very stable in the nucleus and persistently repress the transcription of downstream genes. CRY1-mediated inhibition of Dbp transcription persisted for 24 h in Cry/Per/Nr1d_KO cells after inducing CRY1 nuclear entry (Figures 6B,C). This phenomenon should not be the technical artifact from the fusion of ER to the CRY1 or the addition of 4-OHT. CRY1-mediated Dbp repression could be recovered when exogenous PER2 was expressed in the same cells (Figure 6D). These data also hinted that CLOCK-BMAL1 and CRY could not form a stable feedback loop. PER was required for the displacement of CLOCK-BMAL1 and CLOCK-BMAL1-CRY from DNA to reset the transcription to its basal level.

The mechanism to sustain the circadian rhythm is an exciting topic. Many mathematical models have been proposed from different viewpoints (Kim and Forger, 2012; Pett et al., 2016; Narasimamurthy et al., 2018). In different models, CRY and PER, as the central repressors in TTFL, were included but contributed differently. In the model proposed by Kim and Forger (2012), all PER and CRY forms in the nucleus were considered as the repressors. In this model, the CLOCK-BMAL1 activator became inactive when binding with the CRY-PER repressor. Thus, the stoichiometric balance between the CLOCK-BMAL1 activator and CRY-PER repressor was necessary for circadian timekeeping. Based on recent findings (Ye et al., 2014; Chiou et al., 2016) and the data in this paper, CRY and PER played different roles in the regulation of TTFL. We thought that this model could be refined by adding another repressor complex (CRY-CLOCK-BMAL1). So, the model would include three complexes, including CLOCK-BMAL1 activator, CRY-CLCOK-BMAL1 repressor and PER-CRY-CLOCK-BMAL1 inactive complex). In the model proposed by Pett et al. (2016), two activators (Dbp and Bmal1) and three repressors (Rev-erb-α (Nr1d1), Cry1 and Per2) were included to build the model based on their mutual regulations. Cry1 and Per2 were separated because Cry1, but not Per2, was repressed by NR1D1. In this model, Cry1, Per2 and Nr1d1-mediated repressions of each other were the dominant sources in the oscillation. However, protein interactions between BMAL1, CRY1, or PER2 were not considered in this model. CRY-mediated repression also depends on the levels of PER (removing complex) and CLOCK-BMAL1 (forming complex). PER-mediated regulation also depends on the level of CRY (CRY-dependent activity).

In this paper, we found that PER is required for the reactivation of CRY-mediated transcriptional repression. Thus, PER might regulate circadian rhythm through CRY. In the phosphoswitch model (Narasimamurthy et al., 2018), PER2 was either phosphorylated at the β-TrCP site (S478) or FASP site (S659). PER2 phosphorylated at S478 was rapidly degraded. PER2 phosphorylated at S659 was further phosphorylated at neighboring sites to stabilize. In PER2 S478A mice (Masuda et al., 2020), the period of behavioral rhythm was increased. CRY1 and CRY2 protein levels were also increased in the mutant mice. Although the authors proposed that increased CRY proteins extended the repression of CLOCK-BMAL1-regulated, the promoter binding of CRY or CLOCK-BMAL1 was not analyzed. In another study (Patke et al., 2017), the exon11-deletion mutant of Cry1 identified from Delayed Sleep Phase Disorder patient showed stronger CLOCK-BMAL1 binding affinity but weaker binding on the promoter region. Besides, the CLOCK-BMAL1 displacement activity of PER required its casein kinase binding domain (Ye et al., 2014). Thus, we proposed that the ratio of PER and CRY plays critical roles in the circadian rhythm. The stoichiometric balance between CRY and PER is controlled in multiple layers, including transcription, phosphorylation and protein degradation.

## Data Availability Statement

The datasets presented in this study can be found in online repositories. The names of the repository/repositories and accession number(s) can be found below: https://www.ncbi.nlm.nih.gov/geo/query/acc.cgi?acc=GSE157946.

## Author Contributions

Y-YC designed the experiments, and performed the experiments, data analysis, and manuscript writing. T-YL and YY performed the experiments. AS designed experiments. All authors contributed to the article and approved the submitted version.

## Conflict of Interest

The authors declare that the research was conducted in the absence of any commercial or financial relationships that could be construed as a potential conflict of interest.
